# Treatment-related changes in total hip bone mineral density are applicable to trials of varied study designs and to drugs with differing mechanisms of action: meta-regression results from the FNIH-ASBMR SABRE study

**DOI:** 10.1093/jbmr/zjaf100

**Published:** 2025-07-26

**Authors:** Tatiane Vilaca, Li-Yung Lui, Marian Schini, Susan K Ewing, Austin Thompson, Eric Vittinghoff, Douglas C Bauer, Dennis M Black, Mary L Bouxsein, Richard Eastell

**Affiliations:** Division of Clinical Medicine, University of Sheffield, Sheffield, S57AU, United Kingdom; Research Institute, California Pacific Medical Center, San Francisco, CA 94158, United States; Division of Clinical Medicine, University of Sheffield, Sheffield, S57AU, United Kingdom; Department of Epidemiology and Biostatistics, University of California, San Francisco, San Francisco, CA 94158, United States; Department of Epidemiology and Biostatistics, University of California, San Francisco, San Francisco, CA 94158, United States; Department of Epidemiology and Biostatistics, University of California, San Francisco, San Francisco, CA 94158, United States; Department of Epidemiology and Biostatistics, University of California, San Francisco, San Francisco, CA 94158, United States; Department of Medicine, University of California, San Francisco, San Francisco, CA 94158, United States; Department of Epidemiology and Biostatistics, University of California, San Francisco, San Francisco, CA 94158, United States; Center for Advanced Orthopedic Studies, Beth Israel Deaconess Medical Center, and Department of Orthopedic Surgery, Harvard Medical School, Boston, MA 02215, United States; Division of Clinical Medicine, University of Sheffield, Sheffield, S57AU, United Kingdom

**Keywords:** surrogate, bone mineral density, fracture, osteoporosis treatment, mechanism of action, sequential therapy, meta-regression, randomised control trial, SABRE

## Abstract

Emerging anti-osteoporosis therapies might present varied mechanisms of action and demand active control groups or sequential therapies due to ethical or mechanistic reasons. We previously showed a strong association between treatment-induced changes in total hip BMD (THBMD) at 12 and 24 mo and reduced fracture risk in placebo-controlled trials. We determined the surrogate threshold effect: the minimum THBMD difference (active-placebo) in a trial that would predict a significant reduction in fracture risk in trials. In this analysis, we investigated whether these associations are influenced by drug mechanism of action or trial design, including treatment with an anabolic followed by an antiresorptive compared to active control or placebo. We analyzed individual patient data from 22 randomized, placebo-controlled trials (17 antiresorptive, 3 PTH analogs, 1 odanacatib, and 1 romosozumab placebo-controlled phase), and 3 trials of an anabolic followed by an antiresorptive (1 PTH analog and 2 romosozumab). We established treatment-related differences in THBMD changes, calculated fracture risk reductions for radiologic vertebral and all clinical fractures, and estimated study-level associations between these features via meta-regression. We found consistent associations between treatment-related THBMD changes and fracture risk reduction across different drug mechanisms and trial designs. Among placebo-controlled trials, the *r*^2^ values for vertebral fractures were 0.73 (*p* = .0001) and 0.78 (*p* = .0002) at 24 mo, and 0.59 (*p* = .0003) and 0.70 (*p* = .0007) at 12 mo for all drugs vs only antiresorptive drugs, respectively. Similarly, for all clinical fractures, the *r*^2^ were 0.71 (*p* < .0001) and 0.65 (*p* = .0009) at 24 mo and 0.46 (*p* = .0007) and 0.51 (*p* = .002) at 12 mo for all drugs vs only antiresorptive drugs. For trials of an anabolic followed by an antiresorptive, the association between THBMD change and fracture risk reduction was similar to that for the placebo-controlled monotherapy trials. Our analyses indicate robust associations between treatment-induced THBMD changes and fracture risk reduction across various anti-osteoporosis therapies and trial designs, suggesting that treatment-induced changes in THBMD predict anti-fracture efficacy regardless of drug mechanism or trial design.

## Introduction

The Study to Advance BMD as a Regulatory Endpoint (SABRE) aims to foster the development of new osteoporosis therapies by using a surrogate endpoint to replace fractures in future randomized trials. To this end, SABRE has collected individual patient data (IPD) from over 50 RCTs, including all the trials supporting osteoporosis drugs approved in the U.S. This unique database has allowed us to show that treatment-related changes in total hip BMD are strongly associated with fracture risk reductions across randomized, placebo-controlled trials of osteoporosis therapies.^1^

We also determined the surrogate threshold effect (STE), which corresponds to the magnitude of the treatment effect on the surrogate (ie, THBMD change) over 24 mo that would predict a significant treatment effect on the outcome (ie, a reduction in the risk of fractures).^2^ The FDA is formally considering our proposal to use the treatment-related (placebo-active) difference in BMD percentage change as a surrogate endpoint for fractures as the primary outcome in future trials of new osteoporosis therapies.

However, future trials that would use treatment-related changes in total hip BMD as their primary endpoint may differ from the previous trials we have used in our analyses in 2 critical ways. First, recent trials have used active controls due to ethical concerns, as it became increasingly challenging to conduct placebo-controlled trials of new osteoporosis medications in individuals at high risk for fracture. Second, recent developments in osteoporosis treatments have more commonly tested drugs with anabolic or antiresorptive/anabolic mechanisms of action (MOA). These types of drugs have the potential to increase BMD faster and more substantially than antiresorptive agents and are more likely to be the focus of future drug development.

Here, we will extend our previous work by exploring subsets of studies, including drugs with different MOA (anabolic, mixed anabolic, and antiresorptive and uncoupling antiresorptive). We have also added data from 3 recent trials that tested 2 anabolic agents (abaloparatide and romosozumab) followed by an antiresorptive to consolidate the effects of prior anabolic therapy. In the ACTIVExtend^3^ and FRAME^4^ trials, an initial placebo-controlled period of abaloparatide or romosozumab was followed by an antiresorptive drug (alendronate and denosumab, respectively). Finally, in the ARCH trial, women receiving romosozumab followed by alendronate were compared to those receiving alendronate alone.^5^ Our goal was to assess whether the association between the treatment-related change in BMD and anti-fracture efficacy would be similar in trials with an anabolic followed by an antiresorptive compared to placebo or active control as our prior analyses were based on placebo-controlled monotherapy trials.

## Materials and methods

To evaluate the trial-level relationship between treatment-related differences in percent change in total hip BMD and fracture risk reduction, we used meta-regression methodology, which has previously been described in detail.^1^^,^^6–8^ We used IPD from randomized trials involving various osteoporosis medications and their associated fracture outcomes. Fractures were defined and standardized across all studies. Here, we report results for radiographic vertebral fractures and “all clinical fractures,” which refers to the combination of non-vertebral and clinical vertebral fractures. Radiographic vertebral fractures were identified and defined based on the individual study protocols. To ensure comparability across different DXA devices, total hip BMD values (g/cm^2^) for Lunar and Norland participants were converted to Hologic BMD values using equations provided in Lu et al.^1^

Treatment-related differences in percent changes in THBMD at 12, 18, and 24 mo and log relative risk (RRs) for fractures during the entire study period were calculated for each study. For all clinical fractures, where time to fracture is known, Cox proportional hazard models were utilized to determine the treatment effect on fracture risk reduction within each study, reported as log hazard ratios (log HRs). Conversely, for radiographic vertebral fractures, where exact time to fracture is unknown, logistic regression was employed to estimate the treatment effect on fracture risk reduction, reported as log odds ratios (log ORs). We used weighted linear regression models, whereby each study was weighted by the inverse of its standard error of the log RR for the given fracture outcome, to assess the association between treatment-related difference in THBMD percent change and fracture risk reduction for the 2 sets of analyses described below.

### Mechanism of action analysis in placebo-controlled trials

Since the bulk of the placebo-controlled trials in the SABRE project tested drugs with antiresorptive MOAs, the goal of these analyses was to assess whether our results would have applied to drugs with other MOA, such as anabolic or combined anabolic/antiresorptive MOAs. Ideally, we would conduct a subgroup analysis comparing the meta-regression results for antiresorptive trials vs non-antiresorptive MOAs. However, since the number of non-antiresorptive trials was too small to run subgroup analyses, we ran meta-regressions including the full set of studies (ie, with any mechanism of action), then including just the subset of trials testing antiresorptive drugs. Table 1 lists the placebo-controlled trials included in the full set of studies with any MOA and those included in the subset with antiresorptive MOAs. To be included in these analyses, the trials were required to have BMD measured at the relevant interval (ie, 24 mo [primary analysis] or 12 mo [additional analysis]) and have the required fracture endpoint (ie, radiographic vertebral fracture or any clinical fracture).

**Table 1:** Placebo-controlled studies included in the meta-regression analyses using BMD measurement at 24 and 12 mo. The trials included in the antiresorptive-only analysis are listed under antiresorptive drugs.

	**24 mo**		**12 mo**	
**Study**	**V**	**AC**	**V**	**AC**
**Non-antiresorptive drugs**				
** Odanacatib**				
**LOFT (odanacatib)**^**29**^	X	X	X	X
** PTH analogs**				
**ACTIVE (abaloparatide)**^**9**^			X	X
**TOP (PTH 1-84)**^**27**^			X	X
**FPT (teriparatide)**^**28**^	X	X	X	X
** Romosozumab**				
**FRAME (romosozumab)**^**4**^			X	X
**Antiresorptive drugs**				
** Bisphosphonates**				
**ALN Phase 3 (alendronate)**^**10**^		X		X
**FIT VF (alendronate)**^**11**^	X	X	X	X
**FIT CF (alendronate)**^**12**^	X	X	X	X
**FOSIT (alendronate)**^**13**^				X
**MEN (alendronate)**^**14**^	X		X	
**BONE (ibandronate)**^**15**^	X	X	X	X
**IBAN IV (ibandronate)**^**16**^	X	X	X	X
**VERT-NA (risedronate)**^**17**^	X	X	X	X
**HORIZON PFT (zol)**^**18**^	X	X	X	X
**HORIZON RFT (zol)**^**19**^		X		X
** Estrogen therapy**				
**WHI-E (estrogen)**^**20**^				X
**WHI-E + P (estrogen + prog)**^**21**^				X
** Denosumab**				
**FREEDOM (denosumab)**^**22**^	X	X	X	X
** Selective estrogen receptor modulator (SERM)**				
**GENERATIONS (arzoxifene)**^**23**^	X	X	X	X
**BZA (bazedoxifene)**^**24**^	X	X	X	X
**PEARL (lasofoxifene)**^**25**^	X	X	X	X
**MORE (raloxifene)**^**26**^	X	X	X	X
** Total number of studies**	**14**	**15**	**17**	**21**

Abbreviations: 12 mo, 12 months; 24 mo, 24 months; AC, all clinical; prog, progestin; V, vertebral; X, data included in analyses; Zol, zoledronic acid.

For the 24-mo analysis, there were only 2 placebo-controlled trials of non-antiresorptive drugs: teriparatide and odanacatib. Teriparatide is an anabolic agent, while odanacatib is a formation-sparing antiresorptive agent. Thus, both trials were excluded from the antiresorptive-only analyses. We compared the overall results with all agents to the analysis without these 2 studies for these analyses. To include additional non-antiresorptive agents in the full set of studies, we also analyzed treatment-induced changes in THBMD for BMD measured at 12 mo and reduced fracture risk. This allowed us to include 5 trials of non-antiresorptive drugs: 3 trials of PTH analogs, 1 romosozumab trial, and 1 odanacatib trial. In this analysis, we used the treatment-induced changes in THBMD measured at 12 mo and fracture risk reduction during the entire study period, except for the romosozumab trial,^4^ where the fracture data were restricted to the 12 mo when the trial was placebo-controlled.

The meta-regression results were depicted graphically by plotting back-transformed treatment ORs/HRs against treatment-related differences in THBMD percent changes, with each trial represented by a circle proportional to the number of fractures. Fitted regression lines and 95% prediction limits were added for visual clarity. We visually assessed the consistency of the meta-regression “bubble” plots, including all placebo-controlled trials (any MOA) vs antiresorptive trials only, for the 24-mo and 12-mo analyses.

### Analysis including trials of an anabolic followed by an antiresorptive

**Figure 1:**
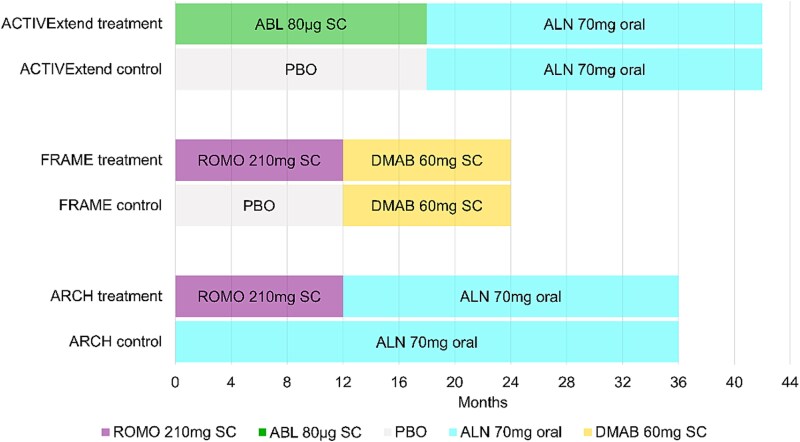
Study-specific treatment regimens for the trials with an anabolic followed by an antiresorptive therapy. Abbreviations: ROMO, romosozumab; ABL, abaloparatide; PBO, placebo; ALN, alendronic acid; DMAB, denosumab; SC, subcutaneous.

Since our primary analyses were limited to monotherapy, placebo-controlled trials,^1^ our goal in these secondary analyses was to assess if the primary meta-regression results could be applied to studies with an anabolic followed by an antiresorptive treatment compared to placebo or active controls. We used data from 3 additional fracture trials to investigate whether treatment-related differences in percent change in THBMD could be used as a surrogate endpoint in these sequential trials. The overall study design of each of the 3 trials is shown in Figure 1.^1^

In the ACTIVExtend trial,^3^ an alendronate open-label extension followed the ACTIVE trial^9^ of abaloparatide compared to placebo. The initial placebo-controlled randomized phase included 1645 women. It lasted 18 mo, followed by a 1-mo no-treatment period (Figure 1). After that, 1139 women who had been randomized to abaloparatide or placebo enrolled in an open-label extension using alendronate (70 mg/weekly) for up to 43 mo after the initial randomization. We used the data from those 1139 participants to estimate the mean percentage change in THBMD in the abaloparatide-treated group over 25 mo (18 mo of abaloparatide or placebo, followed by a 1-mo break followed by 6 mo of alendronate) compared to the “control” group (18 mo of placebo followed by a 1-mo break followed by 6 mo of alendronate). We also calculated the treatment OR for vertebral fracture and the HR for all clinical fracture categories in these 1139 women. As with the placebo-controlled studies, follow-up for fracture outcomes was continued through the end of the study to calculate treatment-related fracture reductions.

The FRAME study^4^ compared romosozumab to placebo for the first 12 mo followed by denosumab treatment in both groups for the subsequent 12 mo (Figure 1). For this sequential treatment study, we used BMD and fracture data over the entire 24-mo follow-up period to calculate the between-group difference in mean percent change in total hip BMD, the treatment OR for vertebral fracture and the treatment HR for all clinical fracture. Since the first 12 mo of FRAME were placebo-controlled, it was included in the 12-mo, but not 24-mo, meta-regression analyses of the placebo-controlled trials described above.

Finally, the ARCH study^5^ compared romosozumab for 12 mo followed by alendronate for 24 mo vs the active control of alendronate for the entire 36 mo in 4093 post-menopausal women (Figure 1). Participants were followed until at least 330 clinical fracture events were confirmed and all participants completed the 24-mo visit. We used the BMD measurements at 24 mo to calculate the between-group difference in mean percentage change in total hip BMD. We also determined treatment-related fracture reductions over the entire treatment period. Since this study was not placebo-controlled, it was not included in any of the meta-regression analyses of the placebo-controlled studies described in the previous section.

Since the number of trials of an anabolic followed by an antiresorptive was too small to run subgroup analyses, we performed meta-regression analyses including all placebo-controlled monotherapy trials plus these three trials. The meta-regression results were depicted graphically by plotting back-transformed treatment ORs/HRs against treatment-related differences in THBMD percent changes, with each trial represented by a circle proportional to the number of fractures. Fitted regression lines and 95% prediction limits were added to the bubble plots. We visually compared these plots to the primary meta-regression plots that included just the placebo-controlled trials.

The STE was defined as the BMD difference (active-placebo) where the upper 95% prediction limit crossed the relative fracture risk of unity. For the STE definition, only placebo-controlled studies were included. For each of the 3 trials of an anabolic followed by an antiresorptive, we checked whether the trial’s difference in the percent change in THBMD between the investigated drug and control at 24 mo exceeded the STEs and, if so, whether the trial reported a nominally significant reduction in fracture risk (*p* < .05).

All analyses were conducted using SAS (version 9.4) and Stata (version 17) software, and all analyses were by intention to treat, without regard to treatment adherence.

## Results

### Mechanisms of action analysis results

In these analyses, we included 122 235 participants from 22 randomized, placebo-controlled trials (Table 1). In the antiresorptive-only analysis, we included 17 trials: 10 bisphosphonates^10–19^; 2 hormone therapies—1 conjugated equine estrogen^20^ and 1 conjugated equine estrogen plus medroxyprogesterone acetate^21^; 1 denosumab^22^; and 4 selective estrogen receptor modulators (SERM).^23–26^ In the “all placebo-controlled studies” analysis, 3 PTH receptor agonist trials,^9^^,^^27^^,^^28^ the first 12 mo of the romosozumab trial (when it was placebo-controlled)^4^ and 1 odanacatib^29^ trial were added. We assessed the trial-level association between treatment-related differences in THBMD percent changes measured at 24 mo (primary analysis) and 12 mo (additional analysis) and fracture risk reduction. The number of trials included in each analysis ranged from 12 to 16 in the “antiresorptive only” analysis and from 14 to 21 in the “all placebo-controlled studies” analysis, depending on the specific fracture type and BMD measurement interval combination.

### Comparison of meta-regression results using all placebo-controlled trials vs antiresorptive trials only, with BMD measurement at 24 mo

**Figure 2:**
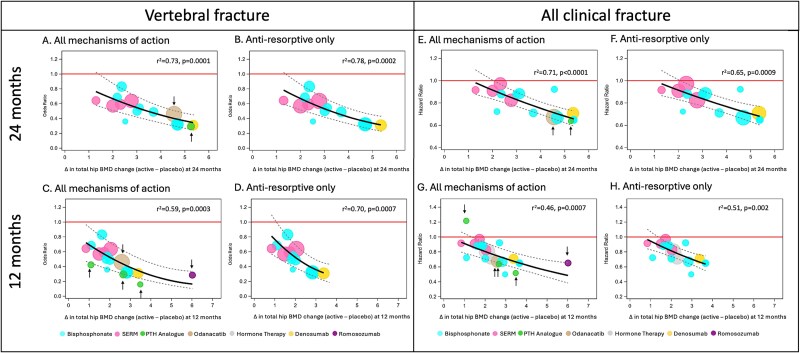
Meta-regression plots for placebo-controlled trials comparing the association of between-treatment difference in THBMD percent change and fracture risk reduction at 24 mo for vertebral fractures for all mechanisms of action (MOA) (A) vs antiresorptive only (B) and 12 mo (C vs D), and the comparison for all clinical fractures at 24 mo (E vs F) and 12 mo (G vs H). The arrows identify non-antiresorptive drugs in the “all mechanisms of action” panels.

Table 2 shows the number of studies, total number of participants, number of participants with fractures, and the meta-regression results for “all placebo-controlled studies” and for “antiresorptive studies only” at 24 mo. Only 1 of the 3 anabolic trials (teriparatide) measured BMD at 24 mo; this trial and the odanacatib trial were added to the “all placebo-controlled studies” analysis. Both trials fell on the meta-regression line in the vertebral fractures and “all clinical fractures” plots (Figure 2A and E).

**Table 2:** Number of placebo-controlled studies, number of participants overall, number of participants with fractures, and meta-regression results for analysis of all mechanisms of action (MOA) and of antiresorptives only at 12 and 24 mo.

	**Vertebral fracture**				**All clinical fracture**			
	**No. Studies**	**No. Participants/No. fractures**	***r*** ^**2**^ **(95% CI)** ***p*-value**	**STE (95% CI)**	**No. Studies**	**No. Participants/No. fractures**	***r*** ^**2**^ **(95% CI)** ***p*-value**	**STE (95% CI)**
**24 mo**								
**All placebo-controlled studies**	14	70 447/4402	0.73 (0.33, 0.84) .0001	1.43 (0.31, 2.46)	15	81 496/7353	0.71 (0.32, 0.83) <.0001	2.04 (1.52, 2.60)
**Antiresorptive studies only**	12	55 130/3406	0.78 (0.37, 0.87) .0002	1.41 (1.29, 2.32)	13	63 792/5987	0.65 (0.19, 0.80) .0009	2.07 (1.70, 5.77)
**12 mo**								
**All placebo-controlled studies**	17	80 235/4573	0.59 (0.19, 0.75) .0003	0.84 (−0.31, 1.56)	21	122 016/11 496	0.46 (0.11, 0.65) .0007	1.37 (0.68, 2.14)
**Antiresorptive studies only**	12	55 130/3406	0.70 (0.23, 0.83) .0007	1.08 (0.85, 1.62)	16	92 955/9831	0.51 (0.11, 0.71) .002	1.38 (1.03, 1.63)

Abbreviations: STE, surrogate threshold effect.

The strength of the association between treatment-related difference in percent change in THBMD and fracture risk reduction at 24 mo was similar for both fracture outcomes when comparing “all placebo-controlled studies” to “antiresorptive studies only” (Table 2). For example, the *r*^2^ for the “all clinical fracture” outcome in the analysis with all studies was 0.71 compared to 0.65 for the antiresorptive studies only, while the STE was 2.04% for all trials vs 2.07% for antiresorptive drugs only.

### Comparison of meta-regression results using all placebo-controlled trials vs antiresorptive trials only, with BMD measurement at 12 mo

In contrast with the 2 non-antiresorptive drug trials included in the 24-mo meta-regression, the analysis of treatment-induced changes in THBMD at 12 mo included 5 non-antiresorptive trials.^8^ Therefore, we explored the analysis of BMD measured at 12 mo, which allowed for a larger set of studies.

Table 2 shows the number of studies, total number of participants, number of participants with fractures, and the meta-regression results for all placebo-controlled studies and for antiresorptive studies only at 12 mo. Figure 2C, D, G, and  H shows the meta-regression plots for the association of between-treatment difference in TH BMD percent change at 12 mo and reduction in vertebral and all clinical fracture risk among all placebo-controlled trials and limited to the antiresorptive trials. Limiting the analysis to the data on THBMD change at 12 mo is unique in allowing the inclusion of 3 anabolic drugs (3 PTH analogs) and 2 drugs with a combined mechanism of action: romosozumab, which has both anabolic and antiresorptive actions, and odanacatib, which has a formation-sparing antiresorptive agent, resulting from a decrease in resorption not followed by a decrease in formation. Therefore, the 12-mo analysis allowed us to include drugs with a range of MOA.

For romosozumab, only data from the first 12 mo of the FRAME study were included, since that is when the trial was placebo-controlled. Compared to placebo, romosozumab showed a larger 1-yr BMD increase (about 6%) than the 3 PTH-analog anabolic drugs (about 1%-3.5%). Despite this greater increase, the romosozumab trial still fell within the meta-regression prediction limits for both vertebral and “all clinical” fracture plots (Figure 2C and G).

The 3 PTH analog studies were relatively small and had a short follow-up, which resulted in wide CIs (Figure S1). In the vertebral fractures plot, all the 3 PTH analog studies fell below the meta-regression line, and the CIs overlap the line. Notably, points below the line would depict a decrease in the risk of fracture greater than the predicted. Therefore, PTH analog trials resulted in a decrease of fracture risk greater than predicted by the model.

All estimates of change exceeded the STE. In the “all clinical fracture” analysis, one study (PTH 1-84) is associated with a HR greater than 1^27^ (Figure 2G). For this study, the increase in THBMD was lower than the STE for all clinical fractures; therefore, our results were consistent with the results observed in the clinical trial. The odanacatib study falls on the line in the vertebral fractures plot (Figure 2C) and below the meta-regression line for “all clinical fracture” (Figure 2G).

The strength of the association between treatment-related difference in change in THBMD and fracture reduction at 12 mo was similar for both fracture outcomes when comparing “all studies” to “antiresorptive only” (Table 2). For vertebral fractures, the *r*^2^ was 0.59 for all therapies vs 0.70 for antiresorptive therapies only, while for all clinical fractures, it was 0.46 and 0.51, respectively. The STEs were also similar for all fracture outcomes when comparing the 2 groups. For vertebral fractures, it was 0.84% for all therapies and 1.08% for antiresorptive only; and for all clinical fractures, it was 1.37% and 1.38%, respectively. Additional 18 mo meta-regression plots for placebo-controlled trials comparing the association of between-treatment difference in TH BMD percent change at 18 months and fracture reduction for vertebral fractures for all mechanisms of action vs. anti-resorptive only and for all clinical fractures for all mechanisms of action vs. anti-resorptive only can be found on supplementary analysis (Supplementary Figure S2 - SF2).

### Anabolic followed by antiresorptive therapy results

To investigate whether the association between treatment-related differences in percent changes in THBMD and fracture risk reduction observed in the monotherapy placebo-controlled trials applies to trials with an anabolic followed by an antiresorptive therapy, we included the 16 placebo-controlled trials and added 3 trials to the 24-mo analysis: ACTIVExtend,^3^ FRAME (24 mo),^4^ and ARCH.^5^ These 19 studies included 94 127 participants.

Table 3 shows the trial-level characteristics for each of the 3 trials, where anabolic therapy was followed by antiresorptive therapy, including sample size, baseline age, baseline total hip BMD, and treatment-related difference in percent change in THBMD and fracture risk reduction. All treatment-related BMD differences were greater than the STE values of 1.43% for vertebral fractures and 2.04% for all clinical fractures. For both fracture outcomes, the between-group differences in THBMD percent change were consistent with significant fracture reductions in FRAME, ARCH, and ACTIVExtend. These results indicate that the STEs for THBMD at 24 mo derived from placebo-controlled monotherapy trials^1^^,^^6^ may be applicable to assess fracture efficacy in trials other than monotherapy compared to placebo.

**Table 3:** Baseline characteristics and treatment-related differences in BMD change and fracture risk reduction for the three trials of an anabolic followed by an antiresorptive.

**Study**	**Total No.**	Baseline Age^*^(years)	Baseline TH BMD^*^ ^**§**^ **(g/cm** ^**2**^ **)**	Active – control difference in 24-mo % change in TH BMD^*^	**Active vs control OR or HR (95% CI)**	
					**Vertebral**	**All clinical**
**ACTIVExtend** ^**3**^	1139	68.6 ± 6.4	0.71 ± 0.09	4.30 ± 0.22	0.15 (0.06, 0.40)	0.66 (0.44, 0.99)
**FRAME** ^**4**^	7180	70.9 ± 7.0	0.64 ± 0.06	5.53 ± 0.11	0.26 (0.16, 0.41)	0.66 (0.51, 0.84)
**ARCH** ^**5**^	4093	74.3 ± 7.5	0.60 ± 0.08	3.81 ± 0.19	0.50 (0.38. 0.64)	0.73 (0.61, 0.88)

Abbreviations: ^*^, mean ± SD; THBMD, total hip BMD; ^§^, standardized baseline BMD.

**Figure 3:**
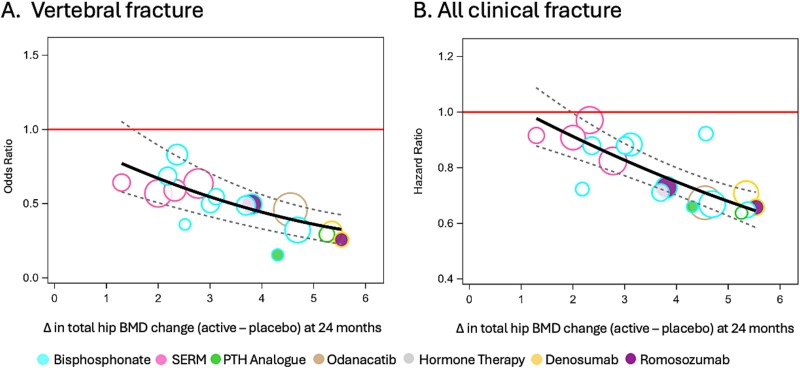
Meta-regression plots for the association of between-treatment difference (group of interest – control**)** in THBMD percent change at 24 mo and fracture risk reduction for vertebral (A) and all clinical (B) fractures. Includes all placebo-controlled trials and 3 trials of an anabolic followed by an antiresorptive.

Table 4 shows the number of studies, number of participants overall, number of participants with fractures and the 24-mo meta-regression results for the placebo-controlled trials and for all trials designs. The *r*^2^ values were similar with or without the 3 trials of an anabolic followed by an antiresorptive: for example, the *r*^2^ for vertebral fracture was 0.73 excluding these 3 trials vs 0.71 when including the 3 trials in the meta-regression. Figure 3 shows the 24-mo meta-regression plots including the 3 trials of an anabolic followed by an antiresorptive. On the plots, the circles for these 3 studies are filled with the colors of the drug of interest, outlined with the colors of the drugs that followed the initial treatment and are marked by arrows. The FRAME and ARCH studies fell on or close to the fitted lines for the 2 fracture types, suggesting that the expected reductions in fracture for a given active-control difference are similar in these 2 studies to the expected value derived from our set of placebo-controlled monotherapy studies. The ACTIVExtend study falls somewhat below the fitted line for the 2 fracture types, suggesting greater reduction in fracture risk than predicted for its between-group difference in THBMD percent change. However, the ACTIVExtend study was relatively small and had wide CIs for HRs that overlapped the regression line. The only exception was for vertebral fracture, where the upper CI bound of the OR was just under the regression line (Figure S3).

**Table 4:** Number of studies, number of participants overall, number of participants with fractures and meta-regression results for placebo-controlled trials and all trial designs at 24 mo.

	**Vertebral fracture**				**All clinical fracture**			
	**No. Studies**	**No. Participants overall**	**No. Participants with fractures**	**r** ^**2**^ **(95% CI)** ***p*-value**	**No. Studies**	**No. Participants overall**	**No. Participants with fractures**	***r*** ^**2**^ **(95% CI)** ***p*-value**
**Placebo- controlled studies**	14	70 447	4402	0.73 (0.33, 0.84) .0001	15	81 496	7353	0.71 (0.32, 0.83) <.0001
**All study designs**	17	81 870	4800	0.71 (0.36, 0.82) <.0001	18	93 908	8177	0.72 (0.39, 0.83) <.0001

## Discussion

Previously, we showed that treatment-related changes in THBMD measured at 12, 18, and 24 mo in placebo-controlled trials are associated with reductions in fracture risk.^1^^,^^8^ Expanding on those findings, here we investigated whether the drug’s mechanism of action and the use of an anabolic followed by an antiresorptive, compared to placebo or antiresorptive control impacted these associations. Among the placebo-controlled trials, we found consistent associations between treatment-related differences in THBMD change and fracture risk reduction at 24 and 12 mo for both vertebral and all clinical fracture outcomes when comparing “all studies” to “antiresorptive trials only.” Furthermore, these associations remained robust when trials of an anabolic followed by an antiresorptive were added to the placebo-controlled 24-mo analyses. Taken together, these results suggest that the use of change in total hip BMD as a surrogate might be used for trials of drugs with varying MOA and for some designs other than placebo-controlled monotherapy.

The predominance of antiresorptive therapies and placebo-controlled trials prevented formal subgroup analyses. There were too few studies to run a meta-regression including only anabolic drugs. Similarly, data were limited to only three trials of an anabolic followed by an antiresorptive. Therefore, our best option for comparing mechanism of action among placebo-controlled trials was to compare the meta-regression results from all studies combined to those from antiresorptive studies only. Our best option for exploring the impact of designs other than placebo-controlled monotherapy was to add those 3 trials to the meta-regression. Notably, each trial’s inclusion in these analyses was driven by the availability of data, that is, the trial had to have IPD for both BMD and fracture outcomes.

Most randomized controlled trials of osteoporosis have tested antiresorptive drugs. Similar to the findings for antiresorptive trials, we showed when non-antiresorptive trials were included in the meta-regression, treatment-related changes in total hip BMD were still associated with a reduction in the risk of fractures. Including drugs with diverse MOA broadens the analysis and enhances the applicability of the SABRE study findings.

Since not all trials measured BMD at the same intervals, we included the additional analysis using BMD measured at 12 mo, which added more studies, increasing the number and range of drugs with non-antiresorptive mechanisms. We also explored using BMD measurements at 18 mo with similar results (Figure S2). The results of the current analyses suggest that our approach can be broadly applied, regardless of drug mechanism of action. Broad applicability is key, as it is likely that new drugs might have anabolic or combined MOAs.

Lastly, to investigate the effect of drugs with varied MOA we included the non-licensed drug odanacatib, an inhibitor of cathepsin K, which has a unique mechanism of action. Odanacatib inhibits the function of osteoclasts while maintaining their vitality. These actions may enable signaling between osteoclasts and osteoblasts, which helps preserve bone formation while inhibiting bone resorption, and so it is referred to as a formation-sparing antiresorptive agent. The uncoupling effects of odanacatib differ from those of other antiresorptive medications, such as bisphosphonates and denosumab, which promote osteoclast apoptosis.^30^ In all plots, the odanacatib trial fell on or very close to the regression line, demonstrating that the meta-regression estimates captured the effects of this drug with its unique mechanism of action.

Despite the different MOA and characteristics of each drug, the *r*^2^ for the association between treatment-related changes in THBMD and fracture risk reduction, and STEs were very similar between the 2 sets of studies. This included drugs that led to a more robust effect on the risk of vertebral fractures, such as PTH analogs; drugs that led to a greater increase in BMD, such as romosozumab; and drugs with a unique mechanism of action, such as odanacatib. These findings highlight the consistency of the association between treatment-related changes in BMD and anti-fracture efficacy across different MOA.

We also explored the impact of trials where anabolic agents were followed by an antiresorptive on the association between treatment-related differences in THBMD changes and fracture risk reduction. The meta-regression results (*r*^2^ and CIs) were very similar in the analyses, including only placebo-controlled trials and after adding trials of an anabolic followed by an antiresorptive. In the plots, these 3 trials fell on or below the regression line. Notably, the FRAME and ACTIVExtend trials tested anabolic agents with a placebo comparison period, followed by a period with treatment with an antiresorptive agent. These trials led to a decrease in vertebral fracture risk greater than the decrease observed in the ARCH study, where romosozumab was compared to an active control from the beginning of the trial. This finding is expected, since the reduction in the risk of fractures in the romosozumb group in the ARCH trial is compared to the active control group, which received alendronate. This active control has an anti-fracture effect itself. All the trials showed a difference in THBMD changes (active-placebo) greater than the STE, consistent with the significant decrease in fracture risk observed in the three trials (Table 3). Notably, the increase in THBMD greater than the STE indicates that the treatment-related increase in BMD is associated with a significant decrease in the risk of fractures but does not quantify the reduction in the risk. The meta-regression included trials with different designs, and the *r*^2^ for the association between treatment-related changes in THBMD and fracture risk reduction was consistent in these scenarios. These results show that the method is robust across different trial designs.

These analyses have a few limitations. We had only a few placebo-controlled trials of non-antiresorptive drugs and only a few trials of an anabolic followed by an antiresorptive, making analyses within these subgroups impossible. The trials for non-antiresorptive medicines were smaller and had a shorter follow-up than the antiresorptive trials. Also, most of the data came from postmenopausal women at increased risk of fracture. All trials enrolled treatment naïve participants; thus, these results may not apply to individuals with prior exposure to osteoporosis therapeutics. Thus, the applicability to other groups is unknown. These limitations should be considered when interpreting the results. Notably, the STE estimates are based on the difference between the active and placebo groups in the percentage change in THBMD and cannot be applied in assessing individual patients in clinical practice.

Conversely, these analyses have several strengths: the SABRE team has gathered a large database with IPD from more than 120 000 participants in rigorous randomized trials. We standardized BMD values and fracture definitions. We included data on drugs from all the categories of MOA currently investigated and trials with varying designs, sizes, and lengths of follow-up.

In summary, trial-level meta-regression analyses using IPD showed that the association between the treatment-induced difference in the change in THBMD and the reduction in fractures was consistent when comparing “all studies” to “antiresorptive studies only” studies. Moreover, this association was also consistent when comparing the meta-regression results based only on placebo-controlled monotherapy studies with meta-regression results including trials of an anabolic followed by an antiresorptive. These observations indicate that the association between treatment-related changes in total hip BMD and anti-fracture efficacy remains strong, regardless of the mechanism of action or trial design. Taken together, these results support the application of total hip BMD change as a surrogate endpoint for future randomized controlled trials of drugs with varied MOA and varied study designs.

## Supplementary Material

SABRE_MOA_seq_supplementary_material_zjaf100

## Data Availability

All study data were acquired by requesting IPD from study sponsors. An overarching data use agreement was created between all parties and individual data use agreements were created between individual study sponsors, FNIH, and University of California, San Francisco (UCSF). Per the data sharing agreements that we have with each sponsor, the data can be used for surrogate marker analyses, including any surrogate qualification processes with regulatory authorities. However, other uses of the data are restricted by this agreement, and UCSF is not allowed to share the data.
