# Supplementary material for: Treatment-related changes in total hip bone mineral density are applicable to trials of varied study designs and to drugs with differing mechanisms of action: meta-regression results from the FNIH-ASBMR SABRE study
Source: J Bone Miner Res. 2025 Jul 26;40(11):1228–37. doi: 10.1093/jbmr/zjaf100 (PMC12578277; doi:10.1093/jbmr/zjaf100)
Supplement: SABRE_MOA_seq_supplementary_material_zjaf100 [file sabre_moa_seq_supplementary_material_zjaf100.docx]

Treatment-related changes in total hip bone mineral density are applicable to trials of varied study designs and to trials of drugs with differing mechanisms of action: meta-regression results from the FNIH-ASBMR SABRE study

Supplementary figure 1


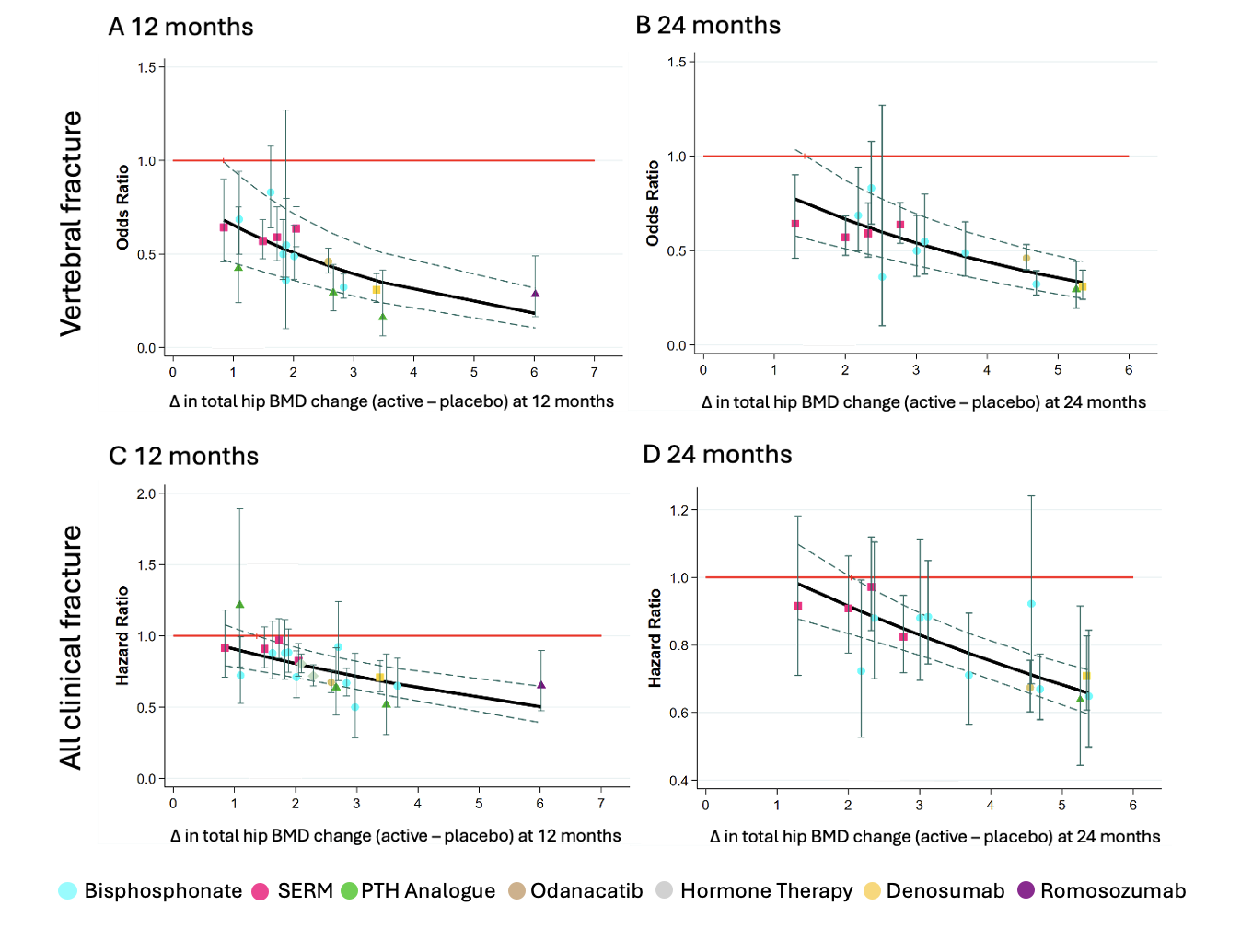


Supplementary figure 1

Meta-regression and confidence interval plots for placebo-controlled trials displaying the association of between-treatment difference in TH BMD percent change and fracture risk reduction for vertebral fracture with BMD measurements at 12 months (A) and 24 months (B) and for all clinical fracture with BMD measurements at 12 (C) and 24 months (D).

Supplementary figure 2


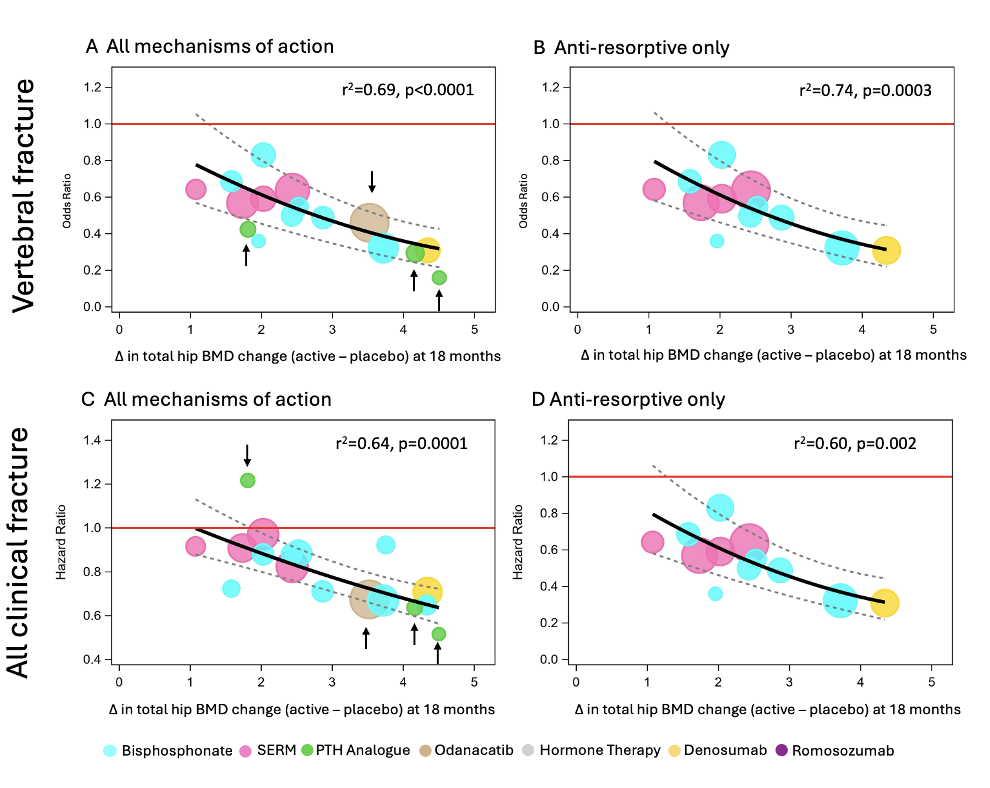


Supplementary figure 2 Meta-regression plots for placebo-controlled trials comparing the association of between-treatment difference in TH BMD percent change at 18 months and fracture reduction for vertebral fractures for all mechanisms of action (A) vs. anti-resorptive only (B) and for all clinical fractures for all mechanisms of action (C) vs. anti-resorptive only (D).

Supplementary figure 3


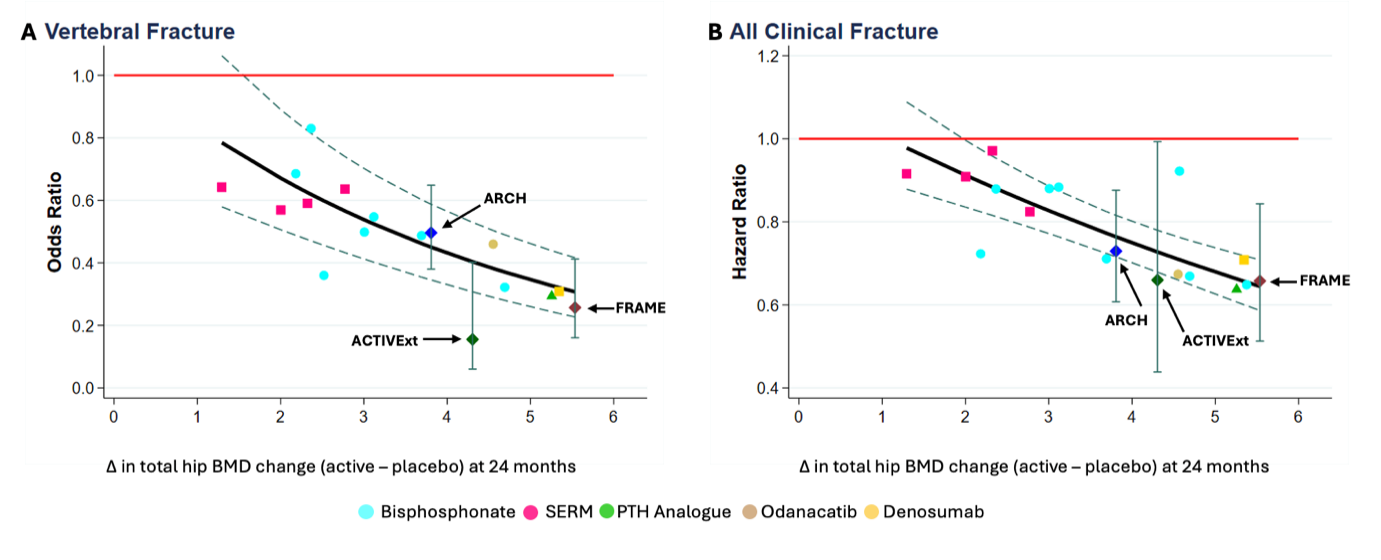


Supplementary figure 3 Meta-regression and confidence interval plots for the association of between-treatment difference in TH BMD percent change at 24 months and fracture risk reduction for vertebral fracture (A) and all clinical fracture (B) including trials with active control or sequential therapy (indicated by the arrows). ACTIVExt: ACTIVExtend Extension trial of the Abaloparatide Comparator Trial In Vertebral Endpoints; FRAME FRActure study in postmenopausal woMen with osteoporosis; ARCH Active‐Controlled Fracture Study in Postmenopausal Women With Osteoporosis at High Risk trial
